# Excess mortality during the COVID-19 pandemic and its spatial distribution in the state of Pernambuco, Brazil: an ecological study

**DOI:** 10.1590/S2237-96222024v33e20231014.en

**Published:** 2024-07-29

**Authors:** Letícia Moreira Silva, Amanda Priscila de Santana Cabral Silva, Maria Helena Rodrigues Galvão, Ana Lúcia Andrade da Silva, Lívia Teixeira de Souza Maia

**Affiliations:** 1 Universidade Federal de Pernambuco, Núcleo de Saúde Coletiva, Vitória de Santo Antão, PE, Brazil

**Keywords:** Excess Mortality, COVID-19, Spatial Analysis, Proportional Mortality Ratio, Ecological Studies, Exceso de Mortalidad, Covid-19, Análisis Espacial, Tasa de Mortalidad Proporcional, Estudios Ecológicos

## Abstract

**Objective:**

To investigate excess mortality during the COVID-19 pandemic and its spatial distribution in Pernambuco, Brazil.

**Methods:**

This was an ecological, descriptive and analytical study of deaths, by municipality, recorded on the Mortality Information System, in 2020 and 2021. Excess mortality was measured by comparing observed and expected deaths, the latter estimated by calculating standardized mortality ratio (SMR). SMR and respective confidence intervals (95%CI) were calculated. Spatial analysis was performed by calculating the Global and Local Moran Index.

**Results:**

Excess mortality was 20.6% and 27.5%, respectively, in 2020 and 2021, with positive spatial correlation (p-value < 0.05). More populous municipalities (2020: SMR = 1.26; 95%CI 1.24;1.27 and 2021: SMR = 1.34; 95%CI 1.32;1.34), more developed municipalities (2020: SMR = 1.43; 95%CI 1.41;1.44 and 2021: SMR = 1.51;95%CI 1.50;1.53) and municipalities in the Sertão region (2020:SMR = 1.31;95%CI 1.30;1.33 and 2021: SMR = 1.44; 95%CI 1.42;1.46) showed greater excess deaths.

**Conclusion:**

Excess mortality coincided with peak periods of COVID-19 transmission.

## Study contributions

**Main results:**

There was excess mortality in Pernambuco in 2020 and 2021, which coincided with the peak of the COVID-19 epidemic curve. Municipalities with higher population density, developed municipalities and municipalities in the Sertão region had higher excess deaths.

**Implications for services:**

The results highlighted the effects of the pandemic on mortality in the state. Strengthening mortality surveillance can assist health management in formulating strategies and public policies for the Brazilian National Health System.

**Perspectives:**

Adoption of this type of analysis could contribute to identification of inequalities in the mortality burden between different population groups, and could be used for analysis of future health challenges.

## RESUMEN

**Objetivo:**

Investigar el exceso de mortalidad durante la pandemia de Covid-19 y su distribución espacial en Pernambuco.

**Métodos:**

Estudio ecológico, descriptivo y analítico de las defunciones, por municipio, registradas en el Sistema de Información de Mortalidad, en 2020 y 2021. El exceso de mortalidad se midió comparando las defunciones observadas y esperadas, estimada por tasas de mortalidad estandarizadas. Se calcularon la SMR y los respectivos intervalos de confianza (IC_95_%). El análisis espacial se realizó mediante el cálculo del Índice de Moran Global y Local.

**Resultados:**

Hubo un exceso de mortalidad del 20,6% y 27,5%, respectivamente, en 2020 y 2021, y una correlación espacial positiva (valor p < 0,05). Municipios más poblados (2020: SMR = 1,26; IC_95_%: 1,24;1,27 y 2021: SMR = 1,34; IC_95_%: 1,32;1,34), más desarrollados (2020: SMR = 1,43; IC_95_%: 1,41;1,44 y 2021: SMR = 1,51; IC_95_%: 1,50;1,53) y Sertão (2020:SMR = 1,31;IC_95_%: 1,30;1,33 y 2021: SMR = 1,44; IC_95_%: 1,42;1,46) presentaron mayor exceso fallecidos.

**Conclusión:**

El exceso de mortalidad coincidió con períodos pico de transmisión de Covid-19.

## INTRODUCTION

The COVID-19 pandemic, compared to the great epidemics in history, such as the 1918 influenza pandemic,^1^ presented itself as one of the biggest global health challenges of this century. The World Health Organization (WHO) estimated that the total number of deaths associated directly or indirectly with the COVID-19 pandemic, between January 1, 2020 and December 31, 2021, was approximately 14.9 million.^2^

Counting cases and deaths is the main means of tracking the growth and trajectory of the pandemic. Notably, measuring excess mortality has proven to be effective in understanding its impact on the world.^3^

There is evidence that the COVID-19 pandemic has caused excess mortality both directly, due to deaths among those infected, and indirectly, due to the lack of demand for health care by individuals fearful of infection, and/or due to the inability of the healthcare system to provide timely and effective services.^4,5^

In Brazil, excess mortality during the COVID-19 period occurred heterogeneously. The North, Northeast and Southeast regions of the country, which recorded higher incidence of COVID-19 cases and deaths, also demonstrated an increase in the number of deaths compared to expected.^6^ In the Northeast, the states of Maranhão, Ceará and Pernambuco were, initially, the main epicenters of the epidemic and concentrated a large part of excess deaths in that region.^6,7^

Occurrence of COVID-19 highlighted the weaknesses in access to health services in Brazil, especially among the lower-income and Black populations. Furthermore, it worsened the economic situation, resulting in increased unemployment, greater income concentration and a reduction in average monthly income. This clearly exposed socio-spatial segregation, as well as the precarious living, working and housing conditions of the most vulnerable groups.^8,9^

Analysis of excess mortality in Pernambuco provides a comprehensive view of the impact of the pandemic on public health in the state. Investigating geographic disparities in excess mortality enables deeper analysis of the factors that contribute to these inequalities.^10,11^ This approach goes beyond analyzing deaths directly related to COVID-19, also encompassing those linked to health conditions and access to services.^5^ This study contributes to understanding the effects of the pandemic, anticipating future challenges and improving the response to public health crises..

The objective of this study was to investigate excess mortality during the COVID-19 pandemic and its spatial distribution in the state of Pernambuco, Brazil. 

## METHODS

We performed a mixed ecological, descriptive and analytical study, based on aggregate data on the mortality of people resident in the municipalities of the state of Pernambuco recorded on the Mortality Information System (Sistema de Informação sobre Mortalidade - SIM), in 2020 and 2021, compared to the average of the five previous years (2015 to 2019).

The state of Pernambuco is located in the Northeast Region of Brazil and had an estimated population of 9,058,931 inhabitants in 2022.^12^ It is made up of 185 municipalities, distributed over 12 Health Regions and five mesoregions: São Francisco Pernambucano; Sertão Pernambucano; Agreste Pernambucano; Mata Pernambucana; Metropolitana do Recife^13^. 

All deaths recorded on the SIM were included in the study, for the periods 2020-2021 and 2015-2019, excluding deaths due to external causes, recorded with cause codes V01 to Y98, according to the International Classification of Diseases 10th revision (ICD-10). 

The study’s dependent variable was excess mortality, defined by the WHO, as a condition in which the number of deaths is higher than expected, according to the mortality pattern previously found in the population.^3^ To this end, the Standardized Mortality Ratio Rate (SMRate) was calculated by municipality and year, using the direct method, considering the age distribution of the state of Pernambuco as a standard and expressed per 100,000 inhabitants. 

The SMRates per municipality, expected for 2020 and 2021, were calculated using the average SMRates between 2015 and 2019. In order to estimate the number of deaths expected for 2020 and 2021 in each of the municipalities, we took the average SMRates calculated for the period 2015-2019 and the population of the municipalities in the respective years. Measurement of excess mortality in 2020 and 2021 was carried out by calculating the absolute and relative differences between the number of deaths found (recorded on the SIM) and expected deaths. 

We also measured the standardized mortality ratio (SMR), based on the quotient between mortality found and mortality expected (standardized). These measures estimate the occurrence of the number of deaths in relation to what would be expected if the municipalities experienced the same rates observed in the previous five-year period. The 95% confidence intervals (95%CI) for each SMR were estimated assuming a Poisson distribution.

In order to analyze excess monthly mortality in 2020 and 2021 in Pernambuco, a control diagram was built so as to be able to compare the number of deaths found with the upper limits (UL) and lower limits (LL) of the number of deaths expected for the period. To this end, we calculated the monthly average number of deaths in the period 2015 to 2019, respective standard deviations (SD) and 95%CI. Thus, the value of expected deaths per month was obtained. In the control diagram, the central line corresponded to average expected deaths (DE), the upper line (UL) refers to the average value + 2SD and the lower line (LL) refers to the average value - 2SD. 

Mortality in the municipalities of Pernambuco was analyzed according to the following characteristics:

I) Population size: 1- up to 20,000 inhab.; 2- 20,001-50,000 inhab.; 3- 50,001-100,000 inhab.; 4- over 100,000 inhab.;
II) Municipal Human Development Index (MHDI): aggregated into four clusters using the K-means method, in ascending MHDI order (C1 to C4);
III) Pernambuco Mesoregions: Agreste, Mata, Metropolitana, São Francisco and Sertão.

Definition of the population size of the municipalities was obtained from estimates made by the Federal Audit Court (Tribunal de Contas da União - TCU) to determine the quotas of the Municipal Participation Fund (Fundo de Participação Municipal - FPM) in 2020.^14^ Data relating to the MHDI were extracted from the Brazilian Human Development Atlas (*Atlas de Desenvolvimento Humano no Brasil*).^15^

We calculated the number of deaths expected, the number of deaths found, excess mortality (absolute and relative), the standardized mortality rate (taxa de mortalidade padronizada – TMP), SMRs and respective 95%CI for the municipalties which were categorized according to the independent variables (size, MHDI and macroregion). 

In the spatial analysis, the Global Moran index was calculated to determine the existence of spatial autocorrelation in the distribution of excess deaths in the state of Pernambuco; the index values ​​vary in intervals of -1 and +1, whereby negative values ​​indicate negative spatial autocorrelation, positive values indicate positive spatial autocorrelation and values ​​close to zero show no spatial pattern.^16^

The Local Moran Index was used to identify spatial autocorrelation of municipalities with excess mortality. This method also made it possible to classify municipalities according to the distribution of their mortality rates in the quadrants of the Moran scatterplot, categorized as: high with neighbor high (1st quadrant), for municipalities that have spatial correlation and have high values; low with neighbor low (2nd quadrant), for municipalities that show spatial correlation and have low values; in addition to two classes of outliers: high with neighbor low (3rd quadrant), for municipalities that showed spatial correlation, but with neighboring municipalities with low values ​​and low with neighbor high (4th quadrant), for municipalities that had spatial correlation with municipalities neighbors with high values.^17^

Data tabulation was performed using Tabwin32 software and the respective definition files (DEF), conversion files (CNV) and data files (DOUFAA.dbc) were generated. Data processing and analysis were carried out using Excel spreadsheets and the Stata 16.1 program, as well as TerraView 4.2.2 to visualize the data through maps representing spatial distribution. 

As this study used public domain data, there were no ethical implications, and submission to a human research ethics committee was not necessary, in accordance with the Resolution No. 466.^18^

## RESULTS

A total of 67,921 deaths were recorded in 2020, resulting in a mortality rate of 7.0/100,000 inhab. Comparing the number of deaths observed that year (n = 11,658) with what was expected for the same period, excess mortality of 20.7% can be seen. In 2021, 72,248 deaths and a mortality rate of 7.4/100,000 inhabitants were recorded, representing excess mortality of 27.7% when compared to what was expected for the period.

**Figure 1:**
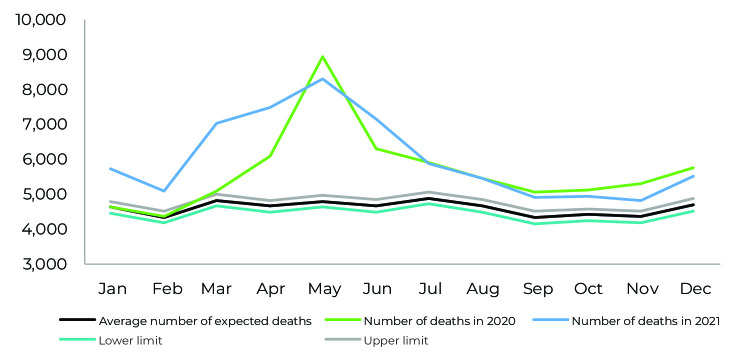
Control diagram of deaths from natural causes by month and year of death, Pernambuco, Brazil, 2020 and 2021

In the period analyzed, excess mortality was highest in May 2020, with 3,968 additional deaths, followed by May and April 2021, with 3,332 and 2,641 additional deaths, respectively. The number of deaths exceeded the upper limit of the epidemic curve from March 2021 onwards, persisting until the end of the period analyzed (Figure 1).

In 2020, there was higher excess mortality among municipalities with the largest population size (Size 4), accounting for 36,259 recorded deaths and an excess of 25.67% (7,405) deaths (SMR = 1.26; 95%CI 1.24;1.27) when compared to smaller sized municipalities. Excess mortality was predominant in municipalities with the highest MHDI, with 28,610 deaths recorded and excess mortality of 42.67% (8,577) (SMR = 1.43; 95%CI 1.41;1.44). Greatest excess mortality was found in the Sertão mesoregion with 21,876 deaths, whereby excess mortality was 31.24% (5,207) (SMR = 1.31; 95%CI 1.30;1.33); and in the Metropolitana macroregion, where 10,526 deaths were recorded and excess mortality was 21.98% (1,897) (SMR = 1.22; 95%CI 1.20;1.24) (Table 1).

**Table 1:** Excess mortality from natural causes in Pernambuco municipalities according to socioeconomic and territorial variables, Pernambuco, Brazil, 2020

Variables	SMRate^b^ (average)	Deaths exepcted	Deaths found	Excess mortality		SMR^c^	95%CI^d^
				**No. of deaths**	**%**		
**Population size**							
Size 1	5.79	5,581	6,479	898	15.01	1.16	(1.13;119)
Size 2	5.89	13,128	14,430	1,302	9.92	1.10	(1.10;1.12)
Size 3	6.28	9,599	10,691	1,092	11.38	1.11	(1.10;1.14)
Size 4	5.89	28,854	36,259	7,405	25.67	1.26	(1.24;1.27)
**MHDI** ^a^							
MHDI - C1	5.46	5,327	5,730	403	7.57	1.10	(1.05;1.10)
MHDI - C2	6.10	14,509	16,368	1,859	12.81	1.13	(1.11;1.15)
MHDI - C3	6.15	15,077	17,151	2,074	13.75	1.14	(1.12;1.16)
MHDI - C4	5.26	20,053	28,610	8,557	42.67	1.43	(1.41;1.44)
**Macroregion**							
Agreste	6.04	15,120	17,585	2,465	16.30	1.16	(1.15;1.18)
Mata	6.14	12,026	14,273	2,247	18.68	1.19	(1.17;1.20)
Metropolitana	5.69	8,629	10,526	1,897	21.98	1.22	(1.20;1.24)
São Francisco	5.39	3,739	3,599	-140	-3.75	0.96	(0.93;0.99)
Sertão	5.66	16,669	21,876	5,207	31.24	1.31	(1.30;1.33)
**Pernambuco**	**6.25**	**56,254**	**67,859**	**11,605**	**20.63**	**1.21**	**(1.20;1.21)**

a) MHDI: Municipal Human Development Index; b) SMRate: Standardized Average Mortality Rate; c) SMR: Standardized Mortality Ratio; d) 95%CI: 95% Confidence Interval.

In Table 2, it can be seen that in 2021 the municipalities with the largest population sizes (Size 4) had a record of 38,802 deaths from natural causes and an excess of 9,753 deaths (33.57%) (SMR = 1.34; 95%CI 1.32;1.34). The municipalities with the highest MHDI had 30,913 deaths and an excess of 10,474 deaths (51.24%) (SMR = 1.51; 95%CI 1.50;1.53). Among the state’s mesoregions, the Sertão and Metropolitan regions continued to have the highest percentages of excess deaths: 7,296 (43.53%) (SMR = 1.44; 95%CI 1.42;1.46) and 2,245 (25.83% ) (SMR = 1.26; 95%CI 1.24;1.28), respectively.

**Table 2:** Excess mortality from natural causes in Pernambuco municipalities according to socioeconomic and territorial variables, Pernambuco, Brazil, 2021

Variables	SMRate^b^ (average)	Expected deaths	Deaths found	Excess mortality		SMR^c^	95%CI^d^
				No. of deaths	%		
**Population size**							
Size 1	5.79	5,598	6,855	1,257	22.46	1.22	(1.96;-1.25)
Size 2	5.89	13,203	15,252	2,049	15.52	1.15	(1.13;-1.17)
Size 3	6.28	9,658	11,267	1,609	16.67	1.16	(1.15;-1.19)
Size 4	5.89	29,049	38,802	9,753	33.57	1.34	(1.32;-1.35)
**MHDI** ^a^							
MHDI - C1	5.46	6,429	7,250	821	12.77	1.13	(1.10;-1.16)
MHDI - C2	6.10	13,859	16,375	2,516	18.16	1.18	(1.64;-1.20)
MHDI - C3	6.15	14,583	17,638	3,055	20.95	1.21	(1.20;-1.23)
MHDI - C4	5.26	20,439	30,913	10,474	51.24	1.51	(1.50;-1.53)
**Macroregion**							
Agreste	6.04	15,214	18,280	3,066	20.15	1.20	(1.18;-1.22)
Mata	6.14	12,077	14,721	2,644	21.89	1.22	(1.20;-1.24)
Metropolitana	5.69	8,689	10,934	2,245	25.83	1.26	(1.24;-1.28)
São Francisco	5.39	3,779	4,183	404	10.69	1.11	(1.10;-1.41)
Sertão	5.66	16,762	24,058	7,296	43.53	1.44	(1.42;-1.46)
**Pernambuco**	**6.25**	**56** **,595**	**72,176**	**15,581**	**27.53**	**1.28**	**(1.27;-1.29)**

a) MHDI: Municipal Human Development Index; b) SMRate: Standardized Average Mortality Rate; c) SMR: Standardized Mortality Ratio; d) 95%CI: 95% Confidence Interval.

**Figure 2:**
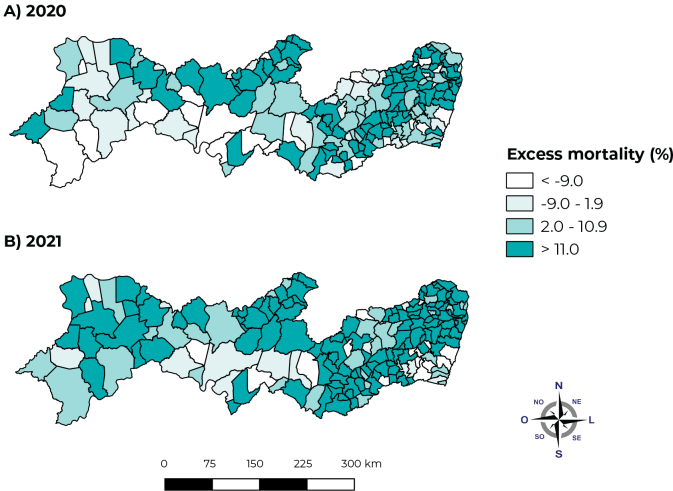
Excess mortality from natural causes by municipality of residence in the state of Pernambuco, Brazil, in 2020 (A) and 2021 (B)

With regard to the spatial analysis of the distribution of deaths due to natural causes and their respective relative percentages of excess mortality, during the study period, it was evident that, in 2020, the Sertão macroregion presented the highest excess deaths (31.24 %), followed by the Metropolitana macroregion, with the second highest excess mortality (21.98%), and by the Agreste macroregion, with 16.3% excess deaths (16.3%) (Figure 2A). 

In 2021, the Sertão and the Metropolitana macroregions continued to have the highest percentages of excess mortality (43.53% and 25.83%, respectively), however, it was possible to observe an increase in percentages between the municipalities of Agreste and Zona da Mata macroregions and a significant increase in the São Francisco marcoregion, revealing the spread of coronavirus into inner regions throughout the state of Pernambuco (Figure 2B).

**Figure 3:**
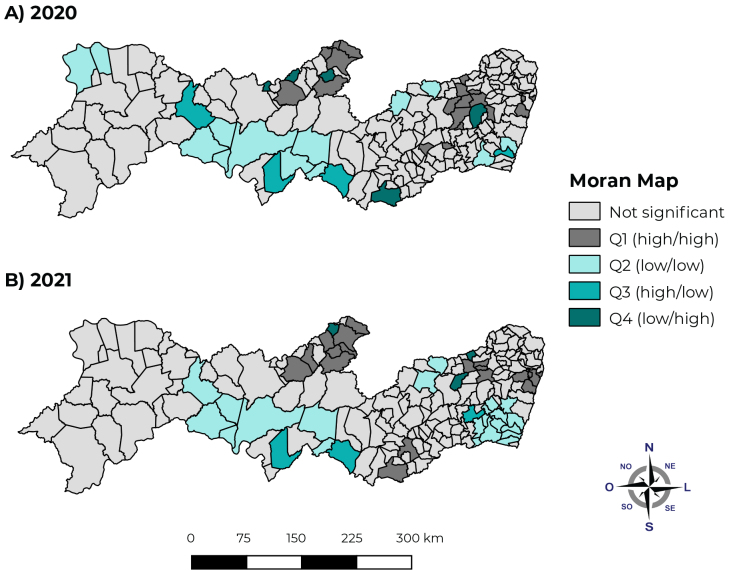
Spatial correlation of excess mortality from natural causes by municipality of residence in the state of Pernambuco, Brazil, in (A) and 2021 (B)

Using the Global Moran Index (I), positive spatial correlation was identified for excess mortality in Pernambuco state in 2020 (I = 0.25; p-value = 0.01) and 2021 (I = 0.30; p-value = 0.01). In 2021, we found the existence of high-high clusters formed by municipalities in the Metropolitana macroregion (Olinda, Recife, Jaboatão dos Guararapes and São Lourenço da Mata), the Agreste marcoregion (Casinhas, Surubim, Limoeiro and João Alfredo) and part of the Sertão macroregion (Brejinho, Itapetim, Santa Terezinha, São José do Egito and Flores) (Figure 3A). In 2022, the presence of high-high clusters present in the Metropolitana, Agreste and Sertão macroregions was persistent, although with a smaller number of municipalities present in these clusters (Figure 3). 

## DISCUSSION

In the period analyzed, there were 27,186 excess deaths, being higher in 2021 (27.7%) and in municipalities with larger population sizes, higher MHDI and concentrated in the Sertão and Metropolitan macroregions. This excess mortality corresponds to the period at the beginning of the COVID-19 pandemic, when the first COVID-19 deaths were recorded in the state. 

During the pandemic, an increase in deaths unrelated to COVID-19 was identified, associated with other infections or disruptions caused by the pandemic,^19^ as well as a reduction in demand for health services due to fear of infection, the collapse of the health system and to the economic impact that influence overall mortality.^20^

A meta-analysis study, which assessed global excess mortality during the COVID-19 pandemic, revealed an aggregate rate of 104.84 deaths per 100,000 inhabitants, highlighting that the number of deaths reported from all causes exceeded expectations on a global scale during the pandemic. Furthermore, a significant increase in excess mortality was observed in regions such as South America, North America, Europe, developing and middle-income countries, as well as among the male population and individuals aged 60 and over.^5^

The results on excess mortality found in this study in Pernambuco are in line with findings from other research carried out at a national level. A study conducted from March to May 2020 in Brazil revealed a 33.5% increase in mortality.^20^ Still in relation to national data, a survey that estimated excess mortality, considering the cause of death, in Brazil as a whole and in the country’s Federative Units (FUs) during the first year of the pandemic, found a 19% increase in deaths.^19^

However, it is crucial to highlight that comprehensive analyses at the national level can mask significant variations within subnational subdivisions, justifying the recommendation for more detailed analyses in the FUs.^19^ This is due to the fact that changes in mortality patterns in Brazil do not occur uniformly, reflecting socio-regional disparities in both exposure to risk factors and accessibility to diagnosis and treatment.^20^

The rates of excess mortality from natural causes, as revealed in this study, showed spatial patterns related to socioeconomic and geographic conditions. Notably, areas with better socioeconomic conditions and larger populations recorded a significant increase in deaths. 

These findings, although they share similarities with other research, also present divergences. A national analysis of excess mortality in 2020 showed a heterogeneous pattern for deaths, correlating positively with areas of greater population density and negatively with the Human Development Index (HDI). Furthermore, it highlighted coincidences in critical periods of the pandemic and a significant increase in deaths from other causes, indicating health system challenges and collapses.^19^

When investigating the process of the spread of COVID-19 into the inner regions of the state of Pernambuco, Quinino et al. (2021) reported higher incidence of COVID-19 in municipalities with higher population density, but with low urbanization rates and lower MHDI. The authors suggest that more populous municipalities favor the spread of the disease.^22^

Research carried out on excess mortality in the state of Minas Gerais in 2020, demonstrated that standardized mortality rates for natural causes showed higher mortality in highly vulnerable sectors, suggesting the existence of social disparities with regard to mortality. 23 Also in Pernambuco, in 2020, a higher number of deaths was identified among municipalities with higher *per* *capita* GDP.^24^

It is important to highlight that estimates of excess mortality are subject to limitations related to analysis and adjustment by age, the latter being a crucial factor to be considered.^25^ Therefore, our calculations were conducted taking into account standardized rates by age, since we sought to make the municipalities of Pernambuco comparable. In the present study, spatial analysis to describe large regions may not have captured local variations, such as neighborhoods, for example. Finally, the demographic data for socioeconomic indicators from the 2010 Census are outdated, which could be a limiting factor. 

The results of this study have expanded the understanding of the possible impacts of COVID-19 on mortality, not only quantifying excess mortality, but also contextualizing these findings in a spatial context in the state of Pernambuco, highlighting the heterogeneity in excess mortality as a reflection of disparities between the state’s municipalities. 

The direct and indirect effects of the pandemic on excess deaths, in both years analyzed were highlighted, possibly related to health services being overloaded, interruption of treatment for chronic diseases and the population’s fear of seeking care due to the risk of infection. This highlights the need for improvements in mortality surveillance and preparation for future public health challenges, especially in municipalities with high mortality rates.

The results reveal that COVID-19 had a direct and indirect impact on mortality, in line with patterns observed in national and international studies. We suggest the implementation of this indicator by health managers, strengthening health surveillance and formulating strategies and policies for future pandemics. We is also recommend that more detailed research be carried out, including analyses by epidemiological week, sex and age group in order to gain a comprehensive understanding of the effects of the pandemic on mortality at state level.
