# Supplementary material for: Excess mortality during the COVID-19 pandemic and its spatial distribution in the state of Pernambuco, Brazil: an ecological study
Source: Epidemiol Serv Saude. 2024 Jul 29;33:e20231014. doi: 10.1590/S2237-96222024v33e20231014.en (PMC11295266; doi:10.1590/S2237-96222024v33e20231014.en)
Supplement: Translation

# Translation: Excesso de mortalidade durante a pandemia de covid-19 e sua distribuição espacial no estado de Pernambuco, um estudo ecológico

**DOI:** 10.1590/S2237-96222024v33e20231014.pt

**Authors:** Letícia Moreira Silva, Amanda Priscila de Santana Cabral Silva, Maria Helena Rodrigues Galvão, Ana Lúcia Andrade da Silva, Lívia Teixeira de Souza Maia

**Affiliations:** 1 Universidade Federal de Pernambuco, Núcleo de Saúde Coletiva, Vitória de Santo Antão, PE, Brasil

**Keywords:** Excesso de Mortalidade, Covid-19, Análise Espacial, Razão de Mortalidade Proporcional, Estudos Ecológicos

## Abstract

**Objetivo:**

Investigar o excesso de mortalidade na pandemia de covid-19 e sua distribuição espacial no estado de Pernambuco.

**Métodos:**

Estudo ecológico, descritivo e analítico dos óbitos, por município, registrados no Sistema de Informação sobre Mortalidade, em 2020 e 2021. O excesso de mortalidade foi mensurado comparando-se os óbitos observados e esperados, este último estimado pelo cálculo das taxas de mortalidade padronizadas. Foram calculados a *standardized mortality ratio* (SMR) e os respectivos intervalos de confiança (IC_95_%). A análise espacial foi realizada pelo cálculo do Índice de Moran Global e Local.

**Resultados:**

Verificou-se excesso de mortalidade de 20,6% e de 27,5%, respectivamente, em 2020 e 2021, e correlação espacial positiva (p-valor < 0,05). Municípios mais populosos (2020: SMR = 1,26; IC_95_% 1,24;1,27 e 2021: SMR = 1,34; IC_95_% 1,32;1,34), mais desenvolvidos (2020: SMR = 1,43; IC_95_% 1,41;1,44 e 2021: SMR = 1,51; IC_95_% 1,50;1,53) e do Sertão (2020: SMR = 1,31; IC_95_% 1,30;1,33 e 2021: SMR = 1,44; IC_95_% 1,42;1,46) apresentaram maior excesso de mortes.

**Conclusão:**

O excesso de mortalidade coincidiu com os períodos de pico de transmissão da covid-19.

## Contribuições do estudo

**Principais resultados:**

Houve excesso de mortalidade em Pernambuco nos anos de 2020 e 2021, que coincidiu com o pico da curva epidêmica de covid-19. Municípios com maior densidade populacional, desenvolvidos e localizados no Sertão apresentaram maior excesso de mortes.

**Implicações para os serviços:**

Os resultados evidenciaram os efeitos da pandemia na mortalidade do estado. O fortalecimento da vigilância da mortalidade pode auxiliar a gestão na formulação de estratégias e políticas públicas para o Sistema Único de Saúde.

**Perspectivas:**

A adoção desse tipo de análise poderá contribuir para a identificação de desigualdades na carga de mortalidade entre diferentes grupos populacionais, podendo ser empregada para análises em futuros desafios sanitários.

## INTRODUÇÃO

A pandemia de covid-19, comparada às grandes epidemias da história, como a influenza de 1918,^1^ apresentou-se como um dos maiores desafios sanitários em escala global deste século. A Organização Mundial da Saúde (OMS) estimou que o número total de mortes associadas direta ou indiretamente à pandemia de covid-19, entre 1º de janeiro de 2020 e 31 de dezembro de 2021, foi de aproximadamente 14,9 milhões.^2^

A contagem de casos e óbitos é o principal meio de rastreamento do crescimento e trajetória da pandemia. Notadamente, a mensuração do excesso de mortalidade vem mostrando-se efetiva para a compreensão do seu impacto no mundo.^3^

Existem evidências de que a pandemia de covid-19 tenha causado excesso de mortalidade tanto de forma direta, em razão dos óbitos entre os infectados, quanto de forma indireta, pela falta de procura ao atendimento de saúde pelos indivíduos com receio de infecção, e/ou devido à incapacidade do sistema de saúde em fornecer serviços oportunos e eficazes.^4,5^

No Brasil, o excesso de mortalidade durante o período da covid-19 se deu de forma heterogênea. As regiões Norte, Nordeste e Sudeste, que registraram maior incidência de casos e óbitos por covid-19, também demonstraram um aumento no número de mortes em relação ao esperado.^6^ No Nordeste, os estados do Maranhão, Ceará e Pernambuco, inicialmente, foram os principais epicentros da epidemia e concentraram grande parte do excesso de óbitos da região.^6,7^

A ocorrência da covid-19 evidenciou as fragilidades no acesso aos serviços de saúde no Brasil, sobretudo entre as populações de menor renda e negra. Além disso, agravou a situação econômica, resultando em aumento do desemprego, maior concentração de renda e redução da média mensal de renda. Isso expôs claramente a segregação socioespacial, as precárias condições de vida, trabalho e moradia dos grupos mais vulneráveis.^8,9^

A análise do excesso de mortalidade em Pernambuco proporciona uma visão abrangente do impacto da pandemia na saúde pública no estado. Investigar disparidades geográficas no excesso de mortalidade possibilita a análise mais aprofundada dos fatores que contribuem para essas desigualdades.^10,11^ Essa abordagem vai além da análise das mortes diretamente relacionadas à covid-19, englobando também aquelas ligadas a condições de saúde e acesso aos serviços.^5^ Esse estudo contribui para a compreensão dos efeitos da pandemia, antecipando desafios futuros e aprimorando a resposta a crises de saúde pública.

Este estudo objetivou investigar o excesso de mortalidade durante a pandemia de covid-19 e sua distribuição espacial em Pernambuco.

## MÉTODOS

Realizou-se um estudo ecológico misto, descritivo e analítico, com base em dados agregados da mortalidade de residentes nos municípios do estado de Pernambuco registrados no Sistema de Informação sobre Mortalidade (SIM), nos anos de 2020 e 2021, comparativamente à média dos cinco anos anteriores (2015 a 2019).

O estado de Pernambuco situa-se na região Nordeste do Brasil e possui uma população estimada de 9.058.931 habitantes no ano de 2022.^12^ É constituído por 185 municípios, distribuídos em 12 Regiões de Saúde e cinco mesorregiões: São Francisco Pernambucano; Sertão Pernambucano; Agreste Pernambucano; Mata Pernambucana; Metropolitana do Recife.^13^

Foram incluídos todos os óbitos registrados no SIM, nos períodos de 2020 a 2021 e de 2015 a 2019, excluindo-se os óbitos por causas externas, registrados com os códigos de causa V01 a Y98, de acordo com a Classificação Internacional de Doenças 10ª revisão (CID-10). 

A variável dependente do estudo foi o excesso de mortalidade, definida pela OMS, como uma condição em que o número de óbitos está situado acima do esperado, segundo o padrão de mortalidade previamente observado na população.^3^ Para tanto, foi calculada a Taxa de Mortalidade Padronizada (TMP) por município e ano, pelo método direto, considerando-se a distribuição etária do estado de Pernambuco como padrão e expressa por 100 mil habitantes. 

As TMPs por município, esperadas para os anos de 2020 e 2021, foram calculadas utilizando-se a TMP média no período de 2015 a 2019. Para estimar o número de óbitos esperados para os anos de 2020 e 2021 em cada um dos municípios, utilizou-se o cálculo da média da TMP no período de 2015 a 2019 e a população dos municípios nos respectivos anos. A mensuração do excesso de mortalidade nos anos de 2020 e 2021 foi realizada calculando-se as diferenças, absoluta e relativa, entre o número de óbitos observados (registrados no SIM) e os óbitos esperados. 

Foi também mensurada a razão de mortalidade padronizada (*standardized mortality ratio* – SMR), a partir do quociente entre a mortalidade observada e a mortalidade esperada (padronizada). Essas medidas estimam a ocorrência do número de óbitos em relação ao que seria esperado se os municípios experimentassem as mesmas taxas observadas no quinquênio anterior. Os intervalos de confiança de 95% (IC_95_%) para cada SMR foram estimados assumindo-se uma distribuição Poisson.

Para análise do excesso de mortalidade mensal nos anos de 2020 e 2021 no estado, foi construído um diagrama de controle, a fim de se comparar o número de óbitos observado com os limites máximo e mínimo do número de óbitos esperados para o período. Para tanto, foi calculada a média mensal do número de óbitos nos anos de 2015 a 2019, os respectivos desvios-padrão (DPs) e IC_95_%. Assim, obteve-se o valor dos óbitos esperados por mês. No diagrama de controle elaborado, a linha central corresponde à média dos óbitos esperados (OE), a linha superior (LS) refere-se ao valor da média + 2DP e a linha inferior (LS) refere-se ao valor da média - 2DP. 

A mortalidade nos municípios pernambucanos foi analisada de acordo com as seguintes características:

I) Porte populacional: 1- até 20.000 hab.; 2- 20.001 a 50.000 hab.; 3- 50.001 a 100.000 hab.; 4- acima de 100.000 hab.;
II) Índice de Desenvolvimento Humano Municipal (IDHM): agregado em quatro *clusters* pelo método K-means, ordenados do IDHM mais baixo ao mais alto (C1 a C4);
III) Mesorregiões do estado: Agreste, Mata, Metropolitana, São Francisco e Sertão.

A definição do porte populacional dos municípios foi obtida a partir de estimativas do Tribunal de Contas da União (TCU) para determinação das cotas do Fundo de Participação dos Municípios (FPM) no ano de 2020.^14^ Os dados relativos ao IDMH foram extraídos do *Atlas de Desenvolvimento Humano no Brasil*.^15^

Foram calculados o número de óbitos esperados, o número de óbitos observados, o excesso de mortalidade (absoluto e relativo), a SMR e os respectivos IC_95_% para os municípios categorizados segundo as variáveis independentes (porte, IDHM e macrorregião). 

Na análise espacial, foi calculado o índice de Moran Global, para determinar a existência de autocorrelação espacial na distribuição do excesso de óbitos no estado de Pernambuco; seus valores variam em intervalos de -1 e +1, os valores negativos indicam autocorrelação espacial negativa, os positivos indicam autocorrelação espacial positiva e os valores próximos a zero evidenciam inexistência de padrão espacial.^16^

O Índice de Moran Local foi utilizado para identificar autocorrelação espacial de municípios com excesso de mortalidade. Este método permitiu igualmente classificar os municípios de acordo com sua distribuição das taxas de mortalidade nos quadrantes do diagrama de espalhamento de Moran, categorizadas como: alto com vizinhança alta (1º quadrante), para os municípios que possuem correlação espacial e têm valores altos; baixo com vizinhança baixa (2º quadrante), para os municípios que apresentam correlação espacial e que possuem valores baixos; além de duas classes de *outliers*: alto com vizinhança baixa (3º quadrante), para os municípios que apresentaram correlação espacial, mas com municípios vizinhos com valores baixos e baixo com vizinhança alta (4º quadrante), para os municípios que possuíam correlação espacial com municípios vizinhos com valores altos.^17^

A tabulação dos dados foi realizada por meio do *software* Tabwin32 e os respectivos arquivos de definição (DEF), conversão (CNV) e de dados (DOUFAA.dbc). O processamento e a análise dos dados foram efetuados por meio de planilhas eletrônicas do Excel e do programa Stata 16.1, além do TerraView 4.2.2 para visualização dos dados através dos mapas de representação da distribuição espacial. 

Por utilizar dados de domínio público, o estudo não apresenta implicações éticas, não sendo necessária a submissão a comitê de ética em pesquisa em seres humanos, de acordo com a Resolução nº 466.^18^

## RESULTADOS

Em 2020, foram registrados 67.921 óbitos, obtendo-se uma TM de 7,0/100 mil hab. Comparando-se o número de óbitos observados nesse ano (n = 11.658) com o esperado para o mesmo período, observa-se um excesso de 20,7%. Em 2021 registraram-se 72.248 óbitos e uma TM de 7,4/100 mil hab., representando um excesso de 27,7% quando comparados com o esperado para o período.

No período analisado, o excesso de mortalidade foi maior no mês de maio de 2020, com 3.968 óbitos adicionais, seguido pelos meses de maio e abril de 2021, com 3.332 e 2.641 óbitos adicionais, respectivamente. O número de óbitos ultrapassou o limite superior da curva epidêmica a partir de março de 2021, persistindo até o final do período analisado (Figura 1).

**Figura 1:** Diagrama de controle dos óbitos por causas naturais segundo mês e ano do óbito, Pernambuco, 2020 e 2021

Em 2020, observou-se maior excesso de mortalidade (EM) entre os municípios de maior porte populacional (Porte 4), com 36.259 óbitos registrados e um excesso de 25,67% (7.405) de óbitos (SMR = 1,26; IC_95_% 1,24;1,27) quando comparados aos municípios de menores portes. O EM foi predominante nos municípios de maior IDHM, com 28.610 óbitos registrados e um excesso de 42,67 % (8.577) de óbitos (SMR = 1,43; IC_95_% 1,41;1,44). Os maiores EMs foram verificados na mesorregião do Sertão, com 21.876 óbitos, um excesso de 31,24% (5.207) de óbitos (SMR = 1,31; IC_95_% 1,30;1,33) e na Metropolitana, onde foram registrados 10.526 óbitos e um excesso de 21,98% (1.897) e (SMR = 1,22; IC_95_% 1,20;1,24) (Tabela 1).

**Tabela 1:** Excesso de mortalidade por causas naturais nos municípios pernambucanos segundo variáveis socioeconômicas e territoriais, Pernambuco, 2020

Variáveis	TMP^b^ (média)	Óbitos esperados	Óbitos observados	Excesso de mortalidade		SMR^c^	IC_95_ **%** ^d^
				**Nº óbitos**	**%**		
**Porte populacional**							
Porte 1	5,79	5.581	6.479	898	15,01	1,16	(1,13;119)
Porte 2	5,89	13.128	14.430	1.302	9,92	1,10	(1,10;1,12)
Porte 3	6,28	9.599	10.691	1.092	11,38	1,11	(1,10;1,14)
Porte 4	5,89	28.854	36.259	7.405	25,67	1,26	(1,24;1,27)
**IDHM** ^a^							
IDHM - C1	5,46	5.327	5.730	403	7,57	1,10	(1,05;1,10)
IDHM - C2	6,10	14.509	16.368	1.859	12,81	1,13	(1,11;1,15)
IDHM - C3	6,15	15.077	17.151	2.074	13,75	1,14	(1,12;1,16)
IDHM - C4	5,26	20.053	28.610	8.557	42,67	1,43	(1,41;1,44)
**Macrorregião**							
Agreste	6,04	15.120	17.585	2.465	16,30	1,16	(1,15;1,18)
Mata	6,14	12.026	14.273	2.247	18,68	1,19	(1,17;1,20)
Metropolitana	5,69	8.629	10.526	1.897	21,98	1,22	(1,20;1,24)
São Francisco	5,39	3.739	3.599	-140	-3,75	0,96	(0,93;0,99)
Sertão	5,66	16.669	21.876	5.207	31,24	1,31	(1,30;1,33)
**Pernambuco**	**6,25**	**56** **.254**	**67.859**	**11.605**	**20,63**	**1,21**	**(1** **,20;1,21)**

a) IDHM: Índice de Desenvolvimento Humano Municipal; b) TMP: Taxa de Mortalidade Média Padronizada; c) SMR: Razão de Mortalidade Padronizada; d) IC_95_%: Intervalo de Confiança de 95%.

Na Tabela 2, verificou-se que em 2021 os municípios de maiores portes populacionais (Porte 4) tiveram um registro de 38.802 óbitos por causas naturais e um excesso de 9.753 mortes (33,57%) (SMR = 1,34; IC_95_% 1,32;1,34). Os municípios de maior IDHM apresentaram 30.913 óbitos e um excesso de 10.474 óbitos (51,24%) (SMR = 1,51; IC_95_% 1,50;1,53). Entre as mesorregiões do estado, Sertão e Metropolitana permaneceram com os maiores percentuais de excesso de óbitos: 7.296 (43,53%) (SMR = 1,44; IC_95_% 1,42;1,46) e 2.245 (25,83%) (SMR = 1,26; IC_95_% 1,24;1,28), respectivamente.

**Tabela 2:** Excesso de mortalidade por causas naturais nos municípios pernambucanos segundo variáveis socioeconômicas e territoriais, Pernambuco, 2021

Variáveis	TMP^b^ (média)	Óbitos esperados	Óbitos observados	Excesso de mortalidade		SMR^c^	IC_95_ **%** ^d^
				**Nº óbitos**	**%**		
**Porte populacional**							
Porte 1	5,79	5.598	6.855	1.257	22,46	1,22	(1,96;-1,25)
Porte 2	5,89	13.203	15.252	2.049	15,52	1,15	(1,13;-1,17)
Porte 3	6,28	9.658	11.267	1.609	16,67	1,16	(1,15;-1,19)
Porte 4	5,89	29.049	38.802	9.753	33,57	1,34	(1,32;-1,35)
**IDMH** ^a^							
IDHM - C1	5,46	6.429	7.250	821	12,77	1,13	(1,10;-1,16)
IDHM - C2	6,10	13.859	16.375	2.516	18,16	1,18	(1,64;-1,20)
IDHM - C3	6,15	14.583	17.638	3.055	20,95	1,21	(1,20;-1,23)
IDHM - C4	5,26	20.439	30.913	10.474	51,24	1,51	(1,50;-1,53)
**Macrorregião**							
Agreste	6,04	15.214	18.280	3.066	20,15	1,20	(1,18;-1,22)
Mata	6,14	12.077	14.721	2.644	21,89	1,22	(1,20;-1,24)
Metropolitana	5,69	8.689	10.934	2.245	25,83	1,26	(1,24;-1,28)
São Francisco	5,39	3.779	4.183	404	10,69	1,11	(1,10;-1,41)
Sertão	5,66	16.762	24.058	7.296	43,53	1,44	(1,42;-1,46)
**Pernambuco**	**6,25**	**56** **.595**	**72.176**	**15.581**	**27,53**	**1,28**	**(1,27;-1,29)**

a) IDHM: Índice de Desenvolvimento Humano Municipal; b) TMP: Taxa de Mortalidade Média Padronizada; c) SMR: Razão de Mortalidade Padronizada; d) IC_95_%: Intervalo de Confiança de 95%.

No que se refere à análise espacial da distribuição dos óbitos por causas naturais e seus respectivos percentuais relativos de excesso de mortalidade, no período do estudo, evidenciou-se que, em 2020, a macrorregião do Sertão apresentou maior excesso de óbitos (31,24%), seguida pela Metropolitana, com o segundo maior excesso de mortalidade (21,98%), e pela região do Agreste, que apresentou um excesso de óbitos (16,3%) (Figura 2A). 

**Figura 2:** Excesso de mortalidade por causas naturais segundo o município de residência no estado de Pernambuco, nos anos de 2020 (A) e 2021 (B)

Em 2021, o Sertão e a Região Metropolitana permaneceram com os maiores percentuais de excesso de mortalidade (43,53% e 25,83%, respectivamente), entretanto, foi possível observar um aumento dos percentuais entre os municípios do Agreste e da Zona da Mata e um aumento significativo na região do São Francisco, revelando a disseminação e interiorização do coronavírus pelo estado de Pernambuco (Figura 2B).

Identificou-se correlação espacial positiva para o excesso de mortalidade no estado nos anos de 2020 (I = 0,25; p-valor = 0,01) e 2021 (I = 0,30; p-valor = 0,01), através do Índice de Moran Global (I). Em 2021 observou-se a existência de *clusters* alto-alto formados por municípios da macrorregião Metropolitana (Olinda, Recife, Jaboatão dos Guararapes e São Lourenço da Mata), do Agreste (Casinhas, Surubim, Limoeiro e João Alfredo) e de parte do Sertão (Brejinho, Itapetim, Santa Terezinha, São José do Egito e Flores) (Figura 3A). Em 2022, foi persistente a presença de *clusters* alto-alto presentes nas macrorregionais Metropolitana, Agreste e Sertão, embora com um menor número de municípios presentes nesses agregados (Figura 3). 

**Figura 3:** Correlação espacial do excesso de mortalidade por causas naturais segundo o município de residência no estado de Pernambuco, em 2020 (A) e 2021 (B)

## DISCUSSÃO

No período analisado, houve excesso de 27.186 óbitos, sendo maior em 2021 (27,7%) e nos municípios com maiores portes populacionais, IDHM e concentrados nas macrorregiões do Sertão e da Metropolitana. Este excesso de mortalidade corresponde ao período de início da pandemia de covid-19, quando foram registradas as primeiras mortes por covid-19 no estado. 

Durante a pandemia, identificou-se crescimento de óbitos não relacionados à covid-19, associados a outras infecções ou interrupções causadas pela pandemia,^19^ como também a redução da procura por serviços de saúde pelo temor da infecção, ao colapso do sistema de saúde e ao impacto econômico que influenciam a mortalidade geral.^20^

Estudo de metanálise, que avaliou o excesso de mortalidade global durante a pandemia de covid-19, revelou uma taxa agregada de 104,84 óbitos por 100 mil hab., destacando que o número de mortes reportadas por todas as causas superou as expectativas em escala mundial durante a pandemia. Além disso, foi observado um aumento significativo do excesso de mortalidade em regiões como América do Sul, América do Norte, Europa, países em desenvolvimento e de renda média, além de entre a população masculina e a de indivíduos com idade igual ou superior a 60 anos.^5^

Os resultados sobre o excesso de mortalidade encontrados neste estudo em Pernambuco estão em consonância com descobertas de outras pesquisas realizadas em nível nacional. Um estudo conduzido nos meses de março a maio de 2020 no Brasil revelou um aumento de 33,5% na mortalidade.^20^ Ainda em relação aos dados nacionais, uma pesquisa que estimou o excesso de mortalidade, considerando a causa do óbito, em todo o Brasil e nas Unidades Federativas (UFs) durante o primeiro ano da pandemia, indicou um acréscimo de 19% nos óbitos.^19^

Entretanto, é crucial ressaltar que análises abrangentes em nível nacional podem mascarar variações significativas dentro das subdivisões subnacionais, justificando a recomendação de análises mais detalhadas nas UF.^19^ Isso se deve ao fato de que as alterações nos padrões de mortalidade no país não ocorrem de modo uniforme, refletindo disparidades sociorregionais tanto na exposição a fatores de risco quanto na acessibilidade a diagnóstico e tratamento.^20^

As taxas de excesso de mortalidade por causas naturais, conforme reveladas neste estudo, apresentaram padrões espaciais relacionados às condições socioeconômicas e geográficas. Notavelmente, áreas com melhores condições socioeconômicas e populações mais numerosas registraram um aumento significativo nas mortes. 

Esses achados, embora compartilhem semelhanças com outras pesquisas, também apresentam divergências. Uma análise nacional do excesso de mortalidade em 2020 mostrou uma heterogeneidade nos óbitos, correlacionando-se positivamente com áreas de maior densidade populacional e negativamente com o Índice de Desenvolvimento Humano (IDH). Além disso, destacou coincidências nos períodos críticos da pandemia e um aumento significativo de óbitos por outras causas, indicando desafios e colapsos nos sistemas de saúde.^19^

Quinino e colaboradores (2021), ao investigarem o processo de interiorização da covid-19 em Pernambuco, relataram maior incidência da covid-19 em municípios com maior densidade populacional**,** porém com baixas taxas de urbanização e menor IDHM. Os autores sugerem que municípios mais populosos favorecem a propagação da doença.^22^

Uma pesquisa realizada sobre o excesso de mortalidade no estado de Minas Gerais, em 2020, demonstrou que as taxas de mortalidade padronizada por causas naturais evidenciaram maior mortalidade em setores de elevada vulnerabilidade, sugerindo a existência de disparidades sociais na mortalidade.^23^ Também em Pernambuco, no ano de 2020, identificou-se maior número de óbitos entre municípios de maior Produto Interno Bruto (PIB) *per capita*.^24^

É relevante destacar que as estimativas de excesso de mortalidade estão sujeitas a limitações relativas à análise e ao ajuste por idade, sendo este último um fator crucial a ser considerado.^25^ Dessa maneira, os cálculos foram conduzidos levando-se em conta as taxas padronizadas por idade, uma vez que se buscou tornar comparáveis os municípios pernambucanos. No presente estudo, a análise espacial para descrever grandes regiões pode não ter capturado as variações locais, como bairros, por exemplo. Por fim, os dados demográficos para os indicadores socioeconômicos do Censo de 2010 estão desatualizados, podendo ser um fator limitante. 

Os resultados deste estudo ampliaram a compreensão sobre os possíveis impactos da covid-19 na mortalidade, não apenas por quantificar o excesso de mortalidade, mas também por contextualizar esses achados em um contexto espacial no estado de Pernambuco, evidenciando a heterogeneidade no excesso de óbitos como um reflexo das disparidades entre os municípios do estado. 

Os efeitos diretos e indiretos da pandemia sobre o excesso de mortes, em ambos os anos analisados, foram destacados, possivelmente relacionados à sobrecarga dos serviços de saúde, interrupção de tratamentos de doenças crônicas e receio da população em buscar atendimento, devido ao risco de infecção. Isso ressalta a necessidade de melhorias na vigilância de óbitos e preparação para futuros desafios em saúde pública, especialmente em municípios com altas taxas de mortalidade.

Os resultados revelam que a covid-19 teve impacto direto e indireto na mortalidade, alinhando-se aos padrões observados em estudos nacionais e internacionais. Sugere-se a implementação desse indicador pelos gestores de saúde, fortalecimento das vigilâncias em saúde e a formulação de estratégias e políticas para pandemias futuras. Recomenda-se ainda a realização de pesquisas mais detalhadas, incluindo análises por semana epidemiológica, sexo e faixa etária, para uma compreensão abrangente dos efeitos da pandemia na mortalidade estadual.
